# Bioluminescence Contributes to the Adaptation of Deep-Sea Bacterium *Photobacterium phosphoreum* ANT-2200 to High Hydrostatic Pressure

**DOI:** 10.3390/microorganisms11061362

**Published:** 2023-05-23

**Authors:** Xu-Chong Bao, Hong-Zhi Tang, Xue-Gong Li, An-Qi Li, Xiao-Qing Qi, Deng-Hui Li, Shan-Shan Liu, Long-Fei Wu, Wei-Jia Zhang

**Affiliations:** 1 Yantai Institute of Coastal Zone Research, Chinese Academy of Sciences, Yantai 264003, China; 2 University of Chinese Academy of Sciences, Beijing 101408, China; 3 Laboratory of Deep-Sea Microbial Cell Biology, Institute of Deep-Sea Science and Engineering, Chinese Academy of Sciences, Sanya 572000, China; 4 Hainan Deep-Sea Technology Laboratory, IDSSE-BGI, Institution of Deep-Sea Life Sciences, Sanya 572000, China; lidenghui@genomics.cn (D.-H.L.); liushanshan@genomics.cn (S.-S.L.); 5 BGI-Qingdao, BGI-Shenzhen, Qingdao 266555, China; 6 LCB, IMM, CNRS, Aix-Marseille University, 13402 Marseille, France

**Keywords:** deep-sea bacterium, bioluminescence, high hydrostatic pressure, oxidative stress, reactive oxygen species

## Abstract

Bioluminescence is a common phenomenon in nature, especially in the deep ocean. The physiological role of bacterial bioluminescence involves protection against oxidative and UV stresses. Yet, it remains unclear if bioluminescence contributes to deep-sea bacterial adaptation to high hydrostatic pressure (HHP). In this study, we constructed a non-luminescent mutant of Δ*luxA* and its complementary strain c-Δ*luxA* of *Photobacterium phosphoreum* ANT-2200, a deep-sea piezophilic bioluminescent bacterium. The wild-type strain, mutant and complementary strain were compared from aspects of pressure tolerance, intracellular reactive oxygen species (ROS) level and expression of ROS-scavenging enzymes. The results showed that, despite similar growth profiles, HHP induced the accumulation of intracellular ROS and up-regulated the expression of ROS-scavenging enzymes such as *dyp*, *katE* and *katG*, specifically in the non-luminescent mutant. Collectively, our results suggested that bioluminescence functions as the primary antioxidant system in strain ANT-2200, in addition to the well-known ROS-scavenging enzymes. Bioluminescence contributes to bacterial adaptation to the deep-sea environment by coping with oxidative stress generated from HHP. These results further expanded our understanding of the physiological significance of bioluminescence as well as a novel strategy for microbial adaptation to a deep-sea environment.

## 1. Introduction

Bioluminescent bacteria are commonly observed in marine habitats, especially deep ocean, where they have been found under either free-living, sessile or symbiotic lifestyles [1]. The majority of luminescent bacteria have been identified in five genera of *Vibrio*, *Photobacterium*, *Aliivibrio*, *Photorhabdus* and *Shewanella*. The molecular mechanism of bacterial bioluminescence has been extensively studied in the model strains of *Aliivibrio fischeri* and *Vibrio campbellii* [2,3,4,5]. As a highly conserved mechanism, luciferase reduces molecular oxygen in water with concomitant oxidation of long-chain aldehydes to long-chain acids. It is an exergonic reaction and the energy is released through light emission with a wavelength of 450–490 nm (blue-green visible light) [6]. Along with this reaction, the reduced flavin mononucleotide (FMNH_2_) is oxidized into oxidized flavin mononucleotide (FMN). The core enzymes required for bioluminescence reaction are encoded by the *lux* gene cluster *luxCDABEG*. The *luxA* and *luxB* encode the α and β subunits of the heterodimeric luciferase, respectively, the LuxCDE forms a fatty acid reductase complex that provides the long-chain aldehyde, and *luxG* encodes a flavin reductase that catalyzes the reduction of FMN to FMNH_2_ using NAD(P)H as an electron donor [7]. This process in shallow water bacteria is under the control of the quorum sensing system LuxIR. When the autoinducer synthesized by LuxI accumulates to a critical concentration, it binds with the receptor LuxR and activates the transcription of the *lux* operon. Therefore, it is assumed that bioluminescence is primarily employed by bacterial communities to synchronize individual behavior. Recently, it has been reported that the emission of bioluminescence by a deep-sea bacterium, *Photobacterium phosphoreum* ANT-2200, is independent of quorum sensing, which suggests a poorly characterized physiological function of bioluminescence in this microorganism [8].

The origin and function of bioluminescence also caught great interest yet remain to be explored. One proposed role of bioluminescence is stimulating DNA repair, possibly through enhanced photoreactivation of photolyase involved in repairing ultra-violet (UV) damaged DNA. Luminescent bacterial cells were more tolerant to UV irradiation than non-luminescent mutants, especially when they were incubated in the dark [9,10]. During a competition experiment, the percentage of luminescent cells outran that of the dark mutant cells when a mixture was applied with UV irradiation [11]. It was thus presumed that photolyase, which requires photons for activation, could be activated by the light generated from bioluminescence. Being capable of conducting DNA repair in a dark environment provides luminescent cells with a growth advantage. Another hypothesis is that luciferase was first evolved to detoxify the deleterious molecular oxygen or to utilize molecular oxygen as an electron acceptor to increase metabolic capacity [12,13]. It was demonstrated that, compared to luminescent *Vibrio* and *Photobacterium*, the growth of non-luminescent mutants was significantly hampered in the presence of oxidants, and the deleterious effect could be significantly reduced by the addition of antioxidants [14,15].

The deep-sea environment is an extreme environment characterized by low temperature, high hydrostatic pressure (HHP) and darkness. It is now known that HHP affects the conformation of macromolecules, enzyme activity and cellular metabolism which can lead to metabolic disorders and redox imbalances and eventually fatality of microorganisms [16,17,18]. Recently, increasing evidence suggested that HHP triggers oxidative stress and antioxidant defense plays a role in microbial adaptation to HHP stress [19,20]. Abram Aertsen et al. first showed that high pressure induces intracellular oxidative stress in *E. coli*, and the mutants deficient in oxidative stress tolerance were more sensitive to elevated pressure [21]. Likewise, oxidative stress resistant bacteria exhibited better tolerance to HHP [22,23]. However, whether bioluminescence, which possibly takes part in anti-oxidation as well, contributes to microbial adaptation to the HHP remains uninvestigated.

In this study, the relationship between bioluminescence and HHP adaptation was analyzed using luminescent strain *P. phosphoreum* ANT-2200. This strain was isolated from the sea-water of the Mediterranean Sea at the depth of 2200 m [24]. It is moderate piezophilic with an optimum growth pressure of 22 MPa and has versatile metabolic capacity [24,25]. Previous studies showed that elevated pressure induced the luminescence of ANT-2200. However, the expression of the *lux* gene cluster was not under the control of pressure nor the well-known quorum-sensing [8]. In this study, we constructed a mutant of Δ*luxA* and its complementary strain c-Δ*luxA* and characterized the involvement of bioluminescence in HHP adaptation through physiological and transcriptomic analyses.

## 2. Materials and Methods

### 2.1. Bacterial Strains

*P. phosphoreum* ANT-2200 was cultured in YPG medium at 25 °C [26]. The cultivation of ANT-2200 and its derivatives were carried out in syringes of 2.5 mL filled with 1.5 mL medium and 1.0 mL air. The syringes were sealed with stoppers before being placed in high-pressure vessels (Feiyu Science and Technology Exploitation Co., Ltd., Nantong, China). The hydrostatic pressure was applied with a water pump (Top Industrie, Vaux-le-Pénil, France). The *luxA* complementary strain (c-Δ*luxA*) was cultured with the supplement of 100 mg/mL kanamycin. The *E. coli* strains were cultured in LB medium at 37 °C with 160-rpm shaking. The diaminopimelic acid (DAP)-auxotroph *E. coli* strain WM3064 (donor strain for bi-parental conjugation) was cultured with the supplementation of 0.3 M of DAP (Sigma-Aldrich, Shanghai, China, catalogue number 33240). The H_2_O_2_ was supplemented to a final concentration of 0.3 mM, unless otherwise indicated. All bacterial strains used in this study are listed in Table 1.

### 2.2. Construction of luxA Mutant and Complementary Strain

To construct a Δ*luxA* mutant, two sequences of 500 bp in length flanking *luxA* were synthesized at GeneWiz Inc. (Suzhou, Jiangsu, China) and introduced into pUX19 at restriction sites of *Xma*I and *Sac*II. The resulting plasmid was introduced into the *P. phosphoreum* ANT-2200 strain by bi-parental conjugation using *E. coli* WM3064 as a donor strain. The *luxA* deletion mutant was screened with a two-steps screening strategy. The first step is to screen for colonies carrying kanamycin resistance cassettes with cultivation on YPG plates containing kanamycin. The second step is to select for double crossover deletion mutant that cannot grow with the presence of 5% sucrose. The potential Δ*luxA* mutant strain was confirmed by PCR amplification and DNA sequencing.

To construct a complementary strain, the DNA fragment containing *luxCDAB* was amplified with forward primer: 5′-TAAAACGACGGCCAGTGAGTTACGAGCTTGGTAAATTCTTTTG-3′, and reverse primer: 5′-AGGAAACAGCTATGACATGATAAAGAAAATCCCAATG-3′ and introduced into board-host vector pBBR1MCS-2 using pEASY-Uni Seamless Cloning and Assembly Kit (TransGen Biotech, Beijing, China, catalogue number CU101-01). The resulting plasmid was transformed into Δ*luxA* through bi-parental conjugation, and colonies with kanamycin resistance were then confirmed by sequencing.

### 2.3. Growth Experiment and Luminescence Assays

Cell samples were collected at different stages of growth. To be specific, 1.0 mL culture was collected for absorption measurement at 600 nm (OD_600 nm_) with Cary 60 UV-Vis spectrophotometer (Agilent Technologies, Santa Clara, CA, USA), and 200 μL culture was collected for luminescence measurement which was carried out using a black, flat-bottom 96-well plate with microplate reader Varioskan LUX (Thermo Fisher Scientific, Waltham, MA, USA). Three replicates were taken for each sample. The average luminescent intensity was divided by the average cell density resulting in the specific luminescent density.

### 2.4. Reactive Oxygen Species (ROS) Quantification

The 2′,7′-dichlorodihydrofluorescein diacetate (DCFH-DA, Sigma-Aldrich, catalogue number D6883) was used to determine the intracellular ROS level. A stock solution (10 mM) was prepared by dissolving 50 mg of DCFH-DA in 10 mL DMSO (Sigma-Aldrich, China, catalogue number D1435). To quantify intracellular ROS, 200 μL culture was taken, and the fluorescent intensity was measured with an excitation of 488 nm and an emission of 526 nm. An average reading (relative fluorescence unit, RFU) was calculated from triplicate measurements. The average reading of the blank medium was subtracted from the average reading of a sample, and then the value was divided by cellular density (OD_600 nm_) and generated the specific fluorescence intensity (RFU/OD_600 nm_).

For the analysis of ROS after short-term incubation, a 15 µM DCFH-DA probe was added into a culture at the early-exponential phase with a cell density of 0.1 OD_600 nm_. The mixture was incubated for one hour under atmospheric pressure, at 25 °C, to allow the diffusion of the probe into bacterial cells. The cells were washed and suspended in fresh YPG medium without the probe before being transferred into syringes and incubated for 2.5 h at different pressure conditions.

To monitor the intracellular ROS during the growth experiment, the DCFH-DA probe was added into the culture to a final concentration of 150 μΜ. The mixture was transferred into syringes and incubated at different pressure conditions. Samples were taken at different growth stages.

### 2.5. RNA Extraction and Real-Time PCR

Total RNA was extracted using Trizol reagent (Ambion, China, catalogue number AM9906) following the manufacturer’s instruction. Briefly, 2.0 mL of mid-exponential phase cultures with a cell density of OD_600 nm_ 0.3 (approximately 10^8^ cells per mL) were centrifuged at 4500× *g* for 30 min. The pellet was homogenized in 1.0 mL of TRIzol, and 200 μL of chloroform was added. After thoroughly shaking and incubation, the samples were centrifuged at 12,000× *g* for 15 min at 4 °C. The upper aqueous phase was transferred to a tube containing an equal volume of isopropanol. The mixtures were thoroughly mixed and incubated at −20 °C for 30 min before centrifuging at 12,000× *g* for 20 min at 4 °C. The pellet was washed in 75% ethanol (prepared by mixing anhydrous ethanol and DEPC-treated water at 3:1) and resuspended in 50 µL DEPC-treated water (Ambion, Shanghai, China, catalogue number AM9906). Extracted RNA samples were treated with RNase-free recombinant DNaseⅠ (TaKaRa, Beijing, China, catalogue number 2270A) at 37 °C for 3 h to remove any residual genomic DNA. The cDNA was synthesized using PrimeScript^TM^ II 1st Strand cDNA Synthesis Kit (TaKaRa, China, catalogue number 6210A). Reaction mixtures with total volumes of 20 µL contained FastStart Universal SYBR Green Master (ROX) (Roche, Mannheim Germany, catalogue number 04913914001), 0.6 µL of each primer (10 mM) and 1 µL of 10-times diluted cDNA as template. The thermal cycling protocol was as follows: initial denaturation for 30 s at 95 °C, followed by 40 PCR cycles of denaturation at 95 °C for 15 s, annealing at 55 °C for 10 s, and extension at 72 °C for 30 s, the final extension lasted for 5 min at 72 °C. Primers used for amplification are shown in Table S1. Reactions were carried out using Applied Biosystems StepOnePlus™ Real-Time PCR (Applied Biosystems, Waltham, MA, USA). All the analyses were performed in triplicates. Relative expression levels were calculated using the *rpoD* gene as the internal reference gene. The relative expression level was calculated as follows: ∆Ct = Ct (gene of interest) − Ct (housekeeping gene), ∆∆Ct = ∆Ct (treated sample) − ∆Ct (untreated sample), Relative expression levels = 2^−(∆∆Ct)^.

### 2.6. HHP Treatment and Growth Experiment

Cells cultured to the early-exponential phase with a cell density of approximately 0.1 OD_600 nm_ were subdivided into two groups and incubated at atmospheric pressure and high hydrostatic pressure (100 MPa) conditions following the method described in Section 2.1. After an incubation of 4 h, cells were collected and diluted 100 times using a fresh medium. The cells were transferred into new syringes and incubated for 30 h in an incubator with light or in the dark by covering the syringe with aluminum foil. Cells were collected at different growth stages and OD_600 nm_ was measured, as described in Section 2.3.

### 2.7. UV Light Irradiation Assay

Cells grown to the mid-exponential phase were collected and diluted to a cell density of 0.05 OD_600 nm_ (approximately 10^7^ cells per mL). Then 20 mL of bacterial cultures were transferred into a clean petri dish and exposed to a UV lamp (8 W) for 5 min. The cells were incubated under light or dark (covered by aluminum foil) conditions for 2 h. For each sample, 100 μL culture was taken for series dilution and spread on YPG plates. The plates were incubated under light or dark conditions at 25 °C for 48 h before the number of colonies was counted. The survival rate was calculated as follows: Survival rate (%) = CFU of UV-treated cell/CFU of non-treated cells × 100%.

## 3. Results

### 3.1. Bioluminescence Favors Bacterial Recovery from High Hydrostatic Pressure

To elucidate the function of bioluminescence in deep-sea bacterium, the *luxA* gene encoding the catalytic subunit of luciferase in the deep-sea bioluminescent strain *P. phosphoreum* ANT-2200 was knocked out, generating a non-luminescent mutant Δ*luxA*. We then introduced a plasmid carrying the *luxCDAB* fragment into the dark mutant and obtained a complementary strain c-Δ*luxA* which is luminescent (Figure S1). The expression of *lux* genes in the three strains was examined by qPCR. As expected, the *luxA* was undetectable in the mutant, but its expression was doubled in the complementary strain compared to the wild-type strain, while expression of other *lux* genes was not affected (Figure S2).

**Figure 1:**
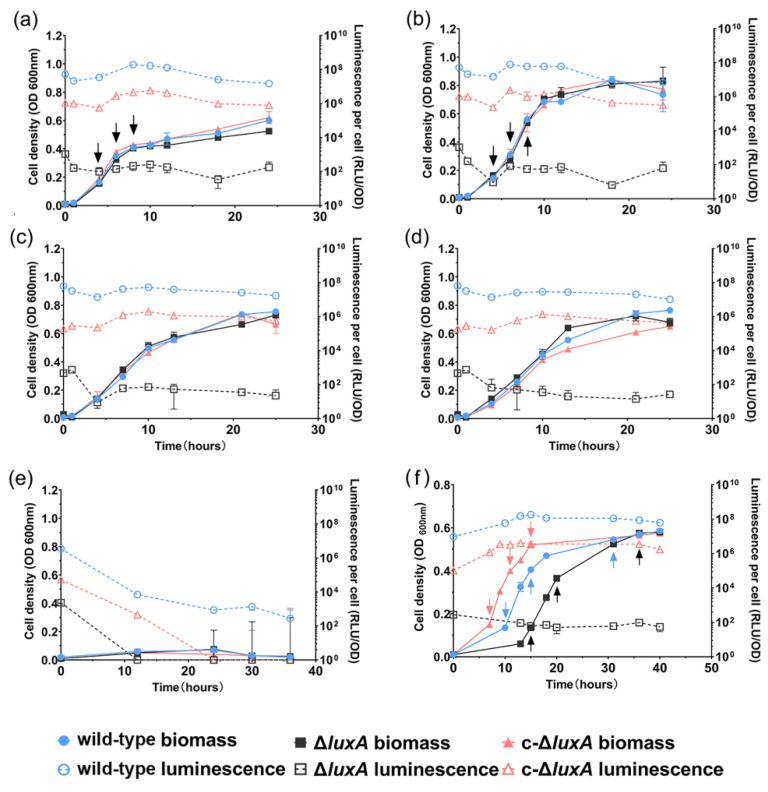
The growth and luminescence characteristics of wild-type strain, Δ*luxA* and c-Δ*luxA* under different pressures. Panels (**a**–**e**) show the growth and luminescence curves at 0.1 MPa, 22 MPa, 30 MPa, 40 MPa and 50 MPa, respectively. Panel (**f**) shows the growth and luminescence curves at 0.1 MPa with the presence of hydrogen peroxide. The solid symbols with solid lines show the cell density indicated by absorption at 600 nm and the hollow symbols with dashed lines show the specific luminescent intensity. Blue, black and red symbols and lines represent wild-type strain, Δ*luxA* mutant strain and c-Δ*luxA* complementary strain, respectively. Arrows in panels (**a**,**b**,**f**) indicate samples collected for quantification of intracellular ROS.

The growth and luminescence of the three strains were examined under pressures of 0.1 MPa, 22 MPa, 30 MPa, 40 MPa and 50 MPa, respectively. The mutant strain was non-bioluminescent by observation. Spectrophotometry analysis confirmed that its bioluminescence intensity was six orders of magnitude lower than the wild-type strain (10^2^ RLU in Δ*luxA* versus 10^8^ in the wild-type strain) (Figure 1). The complementary strain restored luminescence, but its intensity was weaker by approximately an order of magnitude compared to the wild-type strain (10^7^ RLU in c-Δ*luxA* versus 10^8^ in wild-type strain) (Figure 1). Changing pressure from 0.1 MPa to 40 MPa had little influence on the specific luminescence intensity of all three strains. Resembling the wild-type strain, both the Δ*luxA* mutant and the complementary strain c-Δ*luxA* had a higher biomass at 22 MPa than at 0.1 MPa, 30 MPa and 40 MPa. Further increasing the pressure to 50 MPa completely abolished the growth as well as bioluminescence of all three strains.

**Figure 2:**
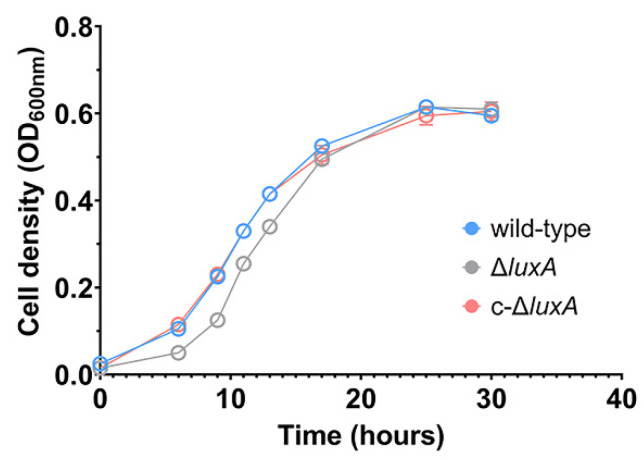
Growth recovery of wild-type strain, Δ*luxA* and c-Δ*luxA* after HHP treatment. The blue, gray and red lines represent wild-type strain, Δ*luxA* mutant and c-Δ*luxA* complementary strain, respectively.

The growth experiment suggested that disruption of bioluminescence did not affect the piezophilic growth phenotype of the deep-sea strain ANT-2200 under pressures below 50 MPa. We then examined the role of bioluminescence under even higher pressures. Considering the fact that pressure over 50 MPa would be lethal for cells of ANT-2200, we cultivated the cells at 0.1 MPa to the early-exponential phase before transferring them to 100 MPa. After incubation for 4 h at 100 MPa, cells were cultivated again at 0.1 MPa. The growth experiment demonstrated that all three strains were able to grow after a treatment of 100 MPa. It was noted that despite a comparable biomass being reached in all three cultures by the late-exponential phase, the Δ*luxA* mutant exhibited a prolonged lag phase compared to the luminescent strains (Figure 2). It seemed that bioluminescence participated in bacterial coping with HHP stress, although the effect appeared only at extremely high pressure under the condition used in this study.

### 3.2. Higher Level of ROS Is Generated by HHP in the Non-Luminescent Mutant

**Figure 3:**
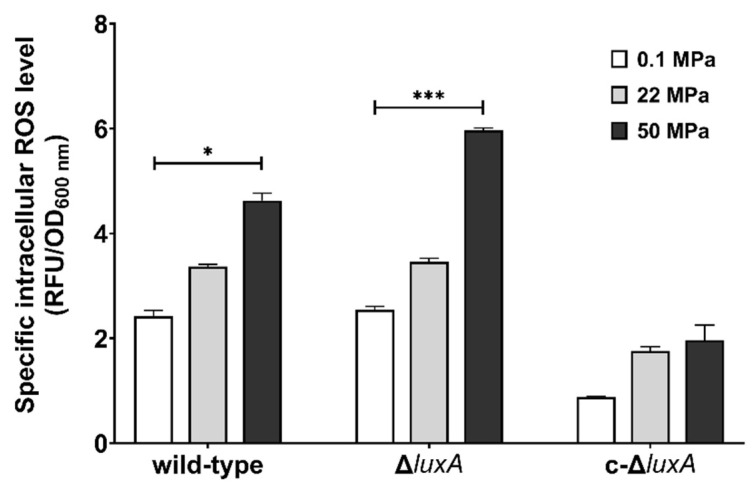
Intracellular ROS levels in the wild-type strain, Δ*luxA*, and c-Δ*luxA* after short-term incubation under different pressures. Specific intracellular ROS level was represented by RFU/OD_600 nm_. The white, grey and black bars represent cultures at 0.1 MPa, 22 MPa and 50 MPa, respectively. Asterisks indicate significant differences: *** *p* < 0.001, * *p* < 0.05 (data were analyzed by one-way ANOVA and the differences among the means were tested using Tukey’s multiple comparison test).

Previous studies in pressure sensitive bacteria and yeast suggested that HHP may lead to oxidative stress [21,27,28]. Meanwhile, molecular oxygen is consumed during bioluminescence, and one potential biological function of microbial bioluminescence is to detoxify molecular oxygen [7]. It was thus presumed that bioluminescence facilitates deep-sea bacterium coping with oxidative stress generated under the HHP condition. To test this hypothesis, we first analyzed the intracellular ROS levels in cells treated with pressures (Figure 3). Cells grown to exponential phase at atmospheric pressure were collected and incubated at different pressures for a short period of 2.5 h before the ROS level was measured. The results showed that compared to cells incubated at 0.1 MPa, raising incubation pressure increased intracellular ROS content in all three strains. When incubated at 50 MPa, the ROS level increased by two times in the wild-type strain and by around three times in the Δ*luxA* mutant strains. Meanwhile, the complementary strain c-Δ*luxA* had the lowest ROS level under all three pressures tested; even incubation at 50 MPa hardly affected its intracellular ROS level.

### 3.3. Alternative ROS-Scavenging Enzymes Are Induced in the Non-Luminescent Mutant in Response to HHP

Elevated pressure resulted in the increase of intracellular ROS content, especially in the non-luminescent cells, indicating that HHP triggers oxidative stress and the level of oxidative stress is dependent on bioluminescence. To corroborate this hypothesis, we examined the expression of ROS-scavenging enzymes in the three strains. A total of seven genes were analyzed, including three genes coding for putative superoxide dismutase (*sodB*, *sod1* and *sod2*), *katE* and *katG* coding for putative catalases, *dyp* coding for Dyp-type peroxidase and *prx* coding for peroxiredoxin C (Table 2).

**Figure 4:**
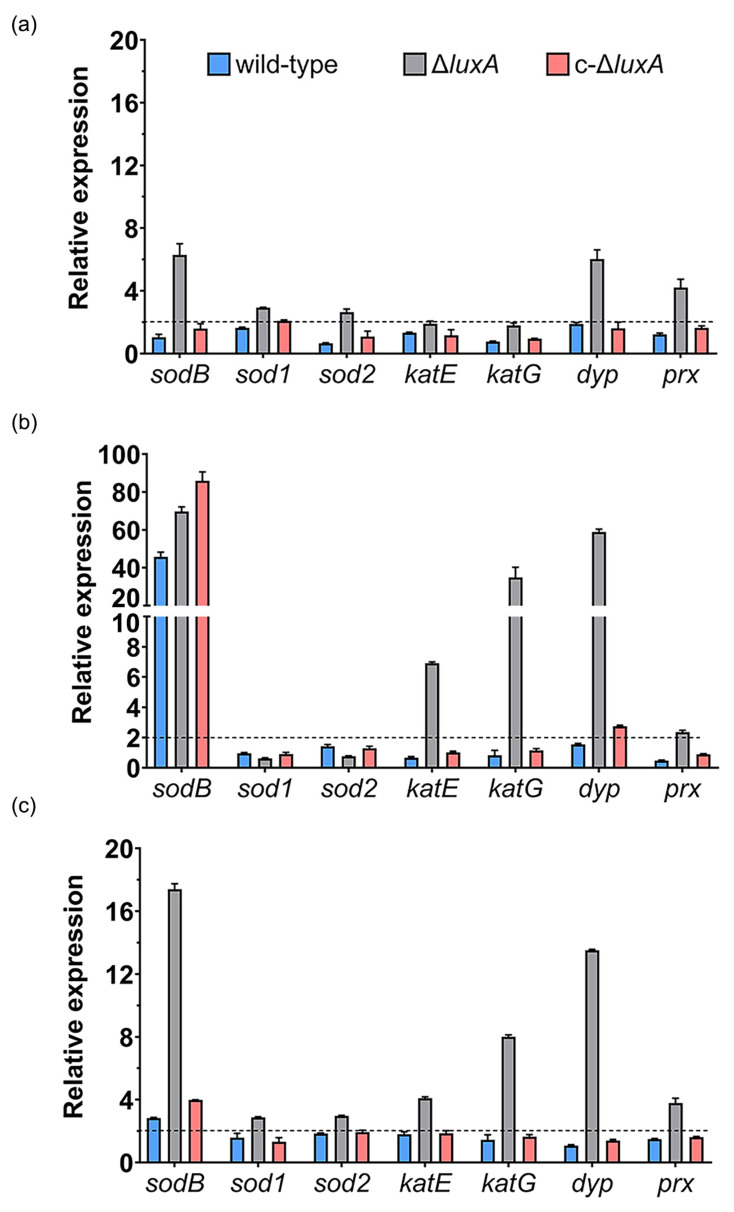
Influence of HHP and H_2_O_2_ on transcription of ROS-scavenging enzymes in the wild-type strain, Δ*luxA* and c-Δ*luxA*. Panels (**a**,**b**) show the relative expression of ROS-scavenging enzymes at 22 MPa and 50 MPa, respectively. The values indicate the abundance of transcripts at elevated pressure relative to atmospheric pressure. Panel (**c**) shows the relative expression of ROS-scavenging enzymes with the presence of 0.3 mM H_2_O_2_. The values indicate the abundance of transcripts in cells grown with H_2_O_2_ relative to cells grown in plain medium at atmospheric pressure. The blue, grey and red bars represent the wild-type strain, Δ*luxA* mutant and complementary strain c-Δ*luxA*, respectively. The dashed lines indicate a 2-fold induction in gene expression.

Compared to cultures at 0.1 MPa, short incubation at 22 MPa up-regulated the expression of *sodB* and *dyp* by over 6-fold, *prx* gene by 4-fold and doubled the expression of the other two superoxide dismutases (*sod1* and *sod2*) in the Δ*luxA*. In contrast, none of these genes was affected in the wild-type strain and the complementary strain (Figure 4a). In cells incubated at 50 MPa, the expression of *sodB* gene was remarkably increased by over 40- to 80-fold in all three strains. In addition, the expression of the two catalases (*katE* and *katG*) and the *dyp* genes were specifically induced in the mutant, while the *dyp* alone was slightly induced in the complementary strain (Figure 4b). To our surprise, the expression of the *prx* gene, which was clearly induced at 22 MPa in the Δ*luxA*, was not affected at 50 MPa.

We further examined the expression of ROS-scavenging enzymes in response to hydrogen peroxide, one of the common sources of oxidative stress. As shown in Figure 4c, *sodB*, *dyp* and *prx* were induced by H_2_O_2_ in the mutant of Δ*luxA*, which was similar to the influence of pressure of 22 MPa, but to a greater level. In addition to that, the two genes coding for catalases were up-regulated by around 4- and 8-fold as well. On the other hand, little influence was observed in the wild-type and the complementary strain, except for *sodB* which was induced by 3- to 4-fold.

### 3.4. Different ROS Elimination Rates in the Luminescent and Non-Luminescent Cells

To further understand how bioluminescence participated in bacterial coping with HHP and oxidative stress, we monitored the changes in ROS levels in the three strains cultured under pressures of 0.1 MPa and 22 MPa, or in the presence of H_2_O_2_ at 0.1 MPa. As presented in Figure 1, all three strains had similar growth profiles at 0.1 MPa and 22 MPa, respectively (Figure 1a,b). In contrast, when grown with the presence of H_2_O_2_, different growth curves were observed. The c-Δ*luxA* strain entered the exponential phase first, followed by the wild-type strain and the Δ*luxA* mutant grew most slowly. It was noted that although the duration of lag phase varied from different strains, same biomass was achieved by the stationary phase (Figure 1f).

The changes of specific ROS levels during growth were examined by sequential sampling. Three samples were collected from each culture for the quantification of the intracellular ROS level: at the early-exponential phase (named as phase I for short) when OD_600 nm_ reached approximately 0.1; the mid-exponential phase (named as phase II) with OD_600 nm_ of approximately 0.3; and the late-exponential phase (named as phase III) with OD_600 nm_ of approximately 0.5 (sampling points are indicated in Figure 1).

**Figure 5:**
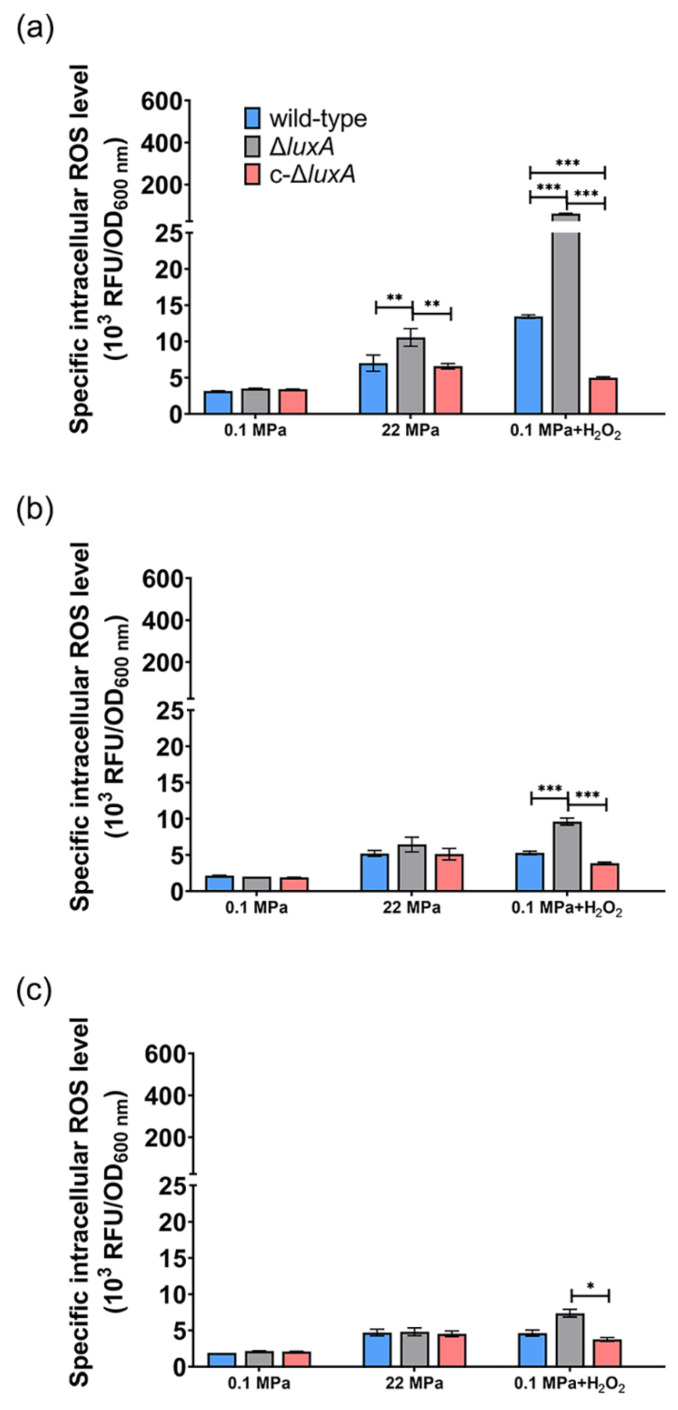
The intracellular ROS level in the wild-type strain, Δ*luxA* and c-Δ*luxA* at different growth stages. Intracellular ROS level was represented by RFU/OD_600nm_. Panels (**a**–**c**), the intracellular ROS level of the three strains at early-exponential phase, mid-exponential phase and late-exponential phase, respectively. Blue, black and red bars and lines represent wild-type strain, Δ*luxA* mutant and c-Δ*luxA* complementary strain, respectively. The sampling points were indicated by arrows in Figure 1. Asterisks indicate significant differences: *** *p* < 0.001, ** *p* < 0.01, * *p* < 0.05 (data were analyzed by one-way ANOVA and the differences among the means were tested using Tukey’s multiple comparison test).

When cultivated at atmospheric pressure, all three strains had comparable ROS levels (Figure 5). The ROS content decreased gradually from around 3 at phase I to around 2 at phase II and remained stable afterward. The application of 22 MPa tripled the intracellular ROS level in the Δ*luxA* mutant and doubled the content in the wild-type and the complementary strain at phase I (22 MPa versus 0.1 MPa). Within 2 h from phase I to phase II, the value decreased from 7.0 to 5.2 in the wild-type strain, from 7.6 to 5.1 in the complementary strain and from 9.5 to 6.5 in the non-luminescent mutant. By phase III, the ROS levels of the three strains were within the range of 4.5 to 4.8, and no significant difference was observed.

Addition of H_2_O_2_ increased the ROS level by 4.3- and 17.5-fold in the wild-type and the Δ*luxA* mutant, respectively, but had little influence on the complementary strain (0.1 MPa + H_2_O_2_ versus 0.1 MPa). The wild-type strain had lower ROS level at phase II than at phase I (5.3 versus 13.4) and stayed stable afterward. Meanwhile, the value in the non-luminescent mutant decreased rapidly from 61.6 to 9.6 during 5 h between phase I and phase II, and kept decreasing during phase II to phase III but at a relatively lower speed. By the stationary phase, the difference between the mutant and the wild-type strain was insignificant.

Taken together, the quantification of ROS at different growth stages confirmed that a pressure of 22 MPa or the presence of hydrogen peroxide induces the accumulation of intracellular ROS, and the effect was more significant in the non-luminescent mutant than in the luminescent cells. The elevated ROS generated from pressure of 22 MPa or H_2_O_2_ could be eliminated during growth, and the Δ*luxA* mutant exhibited a higher ROS decreasing rate, which could be explained by the induced transcription level of ROS-scavenging enzymes.

## 4. Discussion

Previous studies in the deep-sea bacterium *S. piezotolerans* WP3 suggested that adaptation to HHP and oxidative stress are correlated [22]. In this study, by direct quantification of intracellular ROS using fluorescent probe DCFH-DA, we confirmed that incubation under elevated pressure led to oxidative stress in the deep-sea bacterium *P. phosphoreum* ANT-2200. Moreover, compared to the luminescent strains, the induction of ROS by both HHP and hydrogen peroxide was more pronounced in the non-luminescent mutant, especially at the early exponential growth phase.

The qRT-PCR analysis revealed that the two luminescent strains had similar and relatively lower expression levels of antioxidative enzymes, and several kinds of ROS-scavenging enzymes were specifically induced by HHP in the dark mutant. At the optimum pressure of 22 MPa, expression of *sodB* and *dyp* increased most remarkably in the Δ*luxA* mutant. When pressure increased to 50 MPa, the induction of *sodB* was observed in all three strains, while two catalases, a Dyp-type peroxidase and a peroxiredoxin were specifically up-regulated in the dark mutant. The different expression profile of antioxidative enzymes agrees with the changes in ROS content in that, while all three strains were capable of eliminating ROS gradually, the non-luminescent mutant with up-regulated ROS-scavenging enzymes exhibited a higher ROS elimination rate.

Superoxide dismutase, catalase and peroxidase are among the best-characterized antioxidant enzymes. During bacterial oxidative stress defense, superoxide dismutases catalyze the conversion of O_2_^−^ into H_2_O_2_, which are then reduced by catalases and peroxidases into water and oxygen [29]. Peroxiredoxins have similar function as peroxidase, and in addition, participate in H_2_O_2_ sensing and signaling, and maintaining peroxide level [30]. The participation of superoxide dismutase and catalase in bacterial coping with HHP stress has been reported in *E. coli*, *Enterobacter sakazakii*, yeast and deep-sea bacterium *S. piezotolerans* WP3 [22,31,32,33]. Yet, it is the first time that the involvement of peroxidase and peroxiredoxin in HHP adaptation was demonstrated. It should be noted that ROS consists of several kinds of free radicals, such as the superoxide anion (O_2_^−^), singlet oxygen (^1^O_2_), hydroxyl radicals (·OH) and non-radical hydrogen peroxide (H_2_O_2_), etc. The DCFH-DA detects generalized free radicals instead of certain particular ROS [34]. It would be of great interest to identify the nature of ROS molecules generated by HHP and clarify reactions catalyzed by each ROS-scavenging enzyme during bacterial response to HHP. In addition, three putative superoxide dismutases were identified in the genome of ANT-2200; however, only one of them is up-regulated by elevated pressure, indicating different functions among the three iso-enzymes in coping with HHP-induced ROS.

We noticed that despite a relatively lower luminescent intensity, the complementary strain was more tolerant to HHP and oxidative stress than the wild-type strain. The quantification of *lux* genes showed that most of them had comparable expression levels in all three strains, and the only difference lies in the expression of *luxA*, which was doubled in the complementary strain compared to the wild-type strain (Figure S2). Therefore, one possible explanation for the improved tolerance to oxidative stress is that LuxA binds to FMN during a bioluminescence reaction, whose overexpression might alter the intracellular redox homeostasis and enhance the antioxidant capacity through a yet-to-be-discovered mechanism.

**Figure 6:**
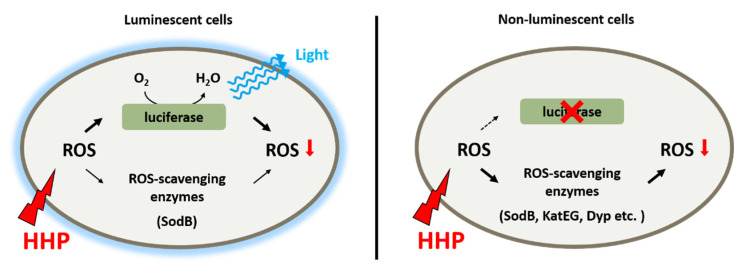
Schematic diagram shows the antioxidant systems in deep-sea bioluminescent bacterium *P. phosphoreum* ANT-2200. Bioluminescence functions as the primary antioxidant system to eliminate ROS generated from HHP. When excess ROS was present, either generated from extremely high pressure or due to impaired bioluminescence, ROS-scavenging enzymes were induced to control the level of intracellular ROS.

Taken together, it seems that bioluminescence participates in the adaptation to HHP by functioning as a primary antioxidant system (Figure 6). As deduced from the slightly increased ROS level and constitutive expression of ROS-scavenging enzymes in the wild-type strain grown under 22 MPa, a functional bioluminescence process would be sufficient to eliminate ROS generated under this condition. Further increased pressure would lead to a higher ROS level and ROS-scavenging enzymes, predominantly SodB, would be induced to cope with the excess ROS molecules. An impaired bioluminescence system induces the expression of several more ROS-scavenging enzymes, such as Dyp-type peroxidase and catalases, indicating their role as an alternative antioxidant system to maintain the homeostasis of intracellular ROS level.

Another possible physiologic function of bioluminescence is to activate DNA photolyase, which stimulates DNA repair by removing the DNA lesions formed by UV such as cyclobutane pyrimidine dimers [9,10,11,35,36]. It was once suggested that light-activated photolyase would be unnecessary in deep-sea microorganisms since sunlight is absent from the deep-sea environment [37]. However, the gene coding for photolyase was identified in the genome of ANT-2200 (PPBDW_v2_II0175), suggesting the capacity of photoreactivation in this deep-sea bacterium. We compared the survival rate of two luminescent (*P. leiognathi* W9 and W214) and a non-luminescent (*P. angustum* QY26) deep-sea *Photobacterium* strains after UV irradiation (Figure S3). The results showed that similar survival rates were obtained for luminescent strains (ANT-2200, W9 and W214), regardless of incubation in the dark or in the light. However, the non-luminescent strain QY26 and the Δ*luxA* mutant of ANT-2200 had a significantly lower survival rate when incubated in the absence of light. We further demonstrated that self-emitted and external light had a similar effect on bacterial recovery from pressurization (Figure S4). The growth of non-luminescent cells was slower than the luminescent ones after treatment at 100 MPa. However, if the cells were grown with exposure to light, same growth profiles were observed in all three strains. This result indicates that, as in shallow water bioluminescent bacteria, photons emitted by luciferase could activate photolyase activity and repair the UV damage in the deep-sea strain ANT-2200. But it remains uncertain if HHP causes similar DNA damage as the UV irradiation does and if the light itself facilitated bacterial recovery from HHP by stimulating photoreactivation.

In summary, we showed in this study that elevated pressure induced oxidative stress in a deep-sea bacterium. Bioluminescent cells could better maintain the ROS level under HHP, while cells with impaired bioluminescence require additional ROS-scavenging enzymes such as catalase and peroxidase to cope with the oxidative stress generated from HHP. Therefore, bioluminescence in the deep-sea bacterium *P. phosphoreum* ANT-2200 takes part in the adaptation to the deep-sea environment as an antioxidant system. These observations expanded our understanding of an alternative strategy in deep-sea bacteria to adapt to the elevated pressure in a deep-sea environment.

## Figures and Tables

**Table 1:** Bacterial strains used in this study.

Strain	Description	Source
*P. phosphoreum* ANT-2200	Isolated from Mediterranean Sea at the depth of 2200 m	[24]
Δ*luxA*	Non-luminescent deficient strains that carrying an interrupted *luxA*	Construct in this study
c-Δ*luxA*	*luxA* deficient strains expressing *luxCDAB* from a board-host plasmid	Construct in this study
*P. leiognathi* W9	Isolated from Mariana Trench 1821 m	Laboratory isolation
*P. leiognathi* W214	Isolated from Mariana Trench 1821 m	Laboratory isolation
*P. angustum* QY26	Isolated from South China Sea 500 m	Laboratory isolation
*E. coli* WM3064	Conjugated transfer donor bacteria, diaminopimelic acid (DAP) deficient	Laboratory stock

**Table 2:** The ROS-scavenging enzymes analyzed in this study.

Name	Locus Tag	Gene ID	Product
*sodB*	PPBDW_RS05155	29942628	Superoxide dismutase [Fe]
*sod1*	PPBDW_RS07830	29944749	Superoxide dismutase family protein
*sod2*	PPBDW_RS16880	29945935	Superoxide dismutase family protein
*katE*	PPBDW_RS16890	29945933	Catalase
*katG*	PPBDW_RS20435	29945712	Catalase
*dyp*	PPBDW_RS18040	29945316	Dyp-type peroxidase
*prx*	PPBDW_RS12520	29943376	Peroxiredoxin C

## Data Availability

All the relevant data related to this study are presented in the manuscript.

## Supplementary Materials

The following supporting information can be downloaded at: https://www.mdpi.com/article/10.3390/microorganisms11061362/s1, Table S1: Primers used for RT-qPCR analyses in this study; Figure S1: The luminescence of colonies of ANT-2200 wild-type, Δ*luxA* mutant and the complementary strain c-Δ*luxA*; Figure S2: The transcription level of *lux* genes in different strains; Figure S3: The influence of UV irradiation on different *Photobacterium* strains. Figure S4: Growth recovery of wild-type strain, Δ*luxA* and c-Δ*luxA* with exposure to light after HHP treatment.
