# Supplementary material for: Bioluminescence Contributes to the Adaptation of Deep-Sea Bacterium Photobacterium phosphoreum ANT-2200 to High Hydrostatic Pressure
Source: Microorganisms. 2023 May 23;11(6):1362. doi: 10.3390/microorganisms11061362 (PMC10304074; doi:10.3390/microorganisms11061362)
Supplement: Supplementary file 1 [file microorganisms-11-01362-s001.zip › microorganisms-2360065-supplementary.pdf]

microorganisms

MDPI

Supplementary materials

Table S1. Primers used for RT-qPCR analyses in this study.

Name	Sequence	Description
sodB-F	TGTCCAGCCTGAACCAAAG	forward primer for amplification of *sodB*
sodB-R	GCAGCTCAAGTATGGAACC	reverse primer for amplification of *sodB*
sod1-F	AACTGTTTTAGGTGGTGCGG	forward primer for amplification of *sod1*
sod1-R	GCATGGATCATTAAAGCGCG	reverse primer for amplification of *sod1*
sod2-F	AGGGCTAGGATGGTCTGAATG	forward primer for amplification of *sod2*
sod2-R	GCTGCAGGTGGTCACTATG	reverse primer for amplification of *sod2*
katE-F	GGGTCTCGCAATGTTTCAGG	forward primer for amplification of *katE*
katE-R	GTATCCCAGAGCGTGTAG	reverse primer for amplification of *katE*
katG-F	AATCGGTTTTGCTGGTGGTC	forward primer for amplification of *katG*
katG-R	GCCGCAAGTAATGGATCTGG	reverse primer for amplification of *katG*
dyp-F	TGCTTTCCGTGGTTCTTTCC	forward primer for amplification of *dyp*
dyp-R	TACCTTCTTCCCACTGTGC	reverse primer for amplification of *dyp*
Prx-F	TCATGATCAAATCCGAGCG	forward primer for amplification of *prx*
Prx-R	AGGGTTTTCTGTGCCATCAAC	reverse primer for amplification of *prx*
luxA-F	CACAAAGATTTCCGCGTTTTTGG	forward primer for amplification of *luxA*
luxA-R	GTCATGCATGTTGGAATTTTC	reverse primer for amplification of *luxA*
luxB-F	GTTGGAGCGATAGCCTTG	forward primer for amplification of *luxB*
luxB-R	CAGTACCAATCGCATGTTG	reverse primer for amplification of *luxB*
luxC-F	AATTTGGGCCGAAGAAAAG	forward primer for amplification of *luxC*
luxC-R	CGCATCAATGTTATCGCC	reverse primer for amplification of *luxC*
luxD-F	TTGAAATCAGAGCATGGTATTG	forward primer for amplification of *luxD*
luxD-R	CATCGAAATCTAAATCTTCTGG	reverse primer for amplification of *luxD*
luxE-F	CGACCTCAATGTTTAAGTATG	forward primer for amplification of *luxE*
luxE-R	TGAACCAAGATATTTCATTCC	reverse primer for amplification of *luxE*
luxF-F	TGTGCTTGCTACCAGTGAAAAC	forward primer for amplification of *luxF*
luxF-R	CTACAGTTTCTGCCTCATTAAC	reverse primer for amplification of *luxF*
luxG-F	AATAGGAATTTGCCCCGTTC	forward primer for amplification of *luxG*
luxG-R	GAGGGCCGCAAACATAG	reverse primer for amplification of *luxG*
rpoD-F	ACGCCGAAGTAAATGACC	forward primer for amplification of *rpoD*
rpoD-R	CGACCGATCTCGCTTTC	reverse primer for amplification of *rpoD*

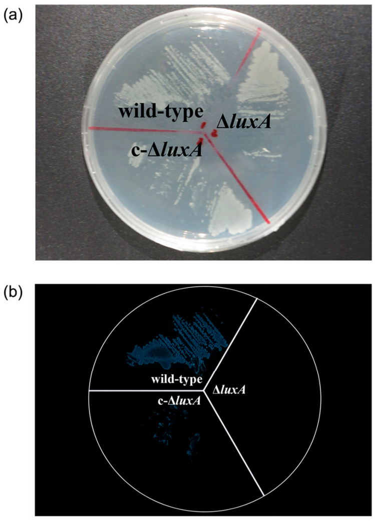

Figure S1. The luminescence of colonies of ANT-2200 wild-type, Δ*luxA* mutant and the complementary strain c-Δ*luxA*. Panel a, the colonies of wild-type, Δ*luxA* and c-Δ*luxA* on YPG plate, photo taken under light condition. Panel b, the luminescence of wild-type, Δ*luxA* and c-Δ*luxA* colonies on YPG plate, photo taken under dark condition.

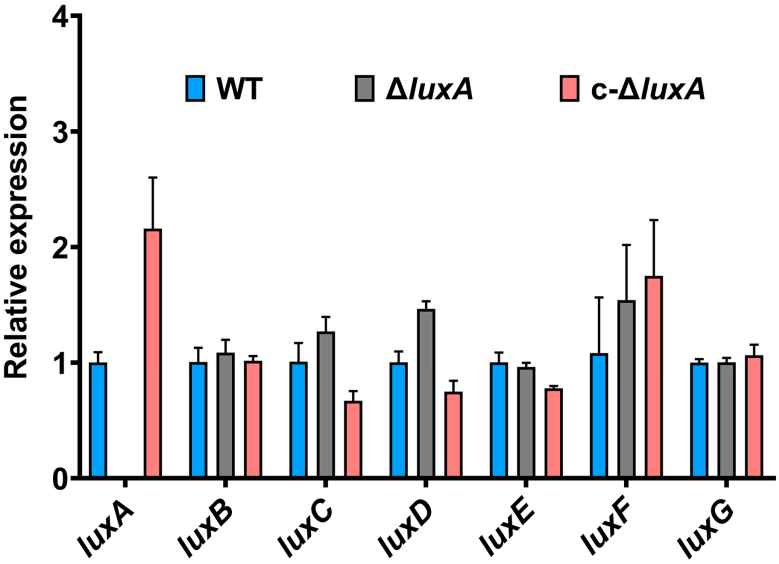

Figure S2. The transcription level of *lux* genes in different strains. The transcription of *lux* genes in different strains relative to the wild-type strain. The *rpoD* gene was used as the internal reference. The blue, gray and red bars represent wild-type strain, Δ*luxA* mutant strain and c-Δ*luxA* complementary strain, respectively.

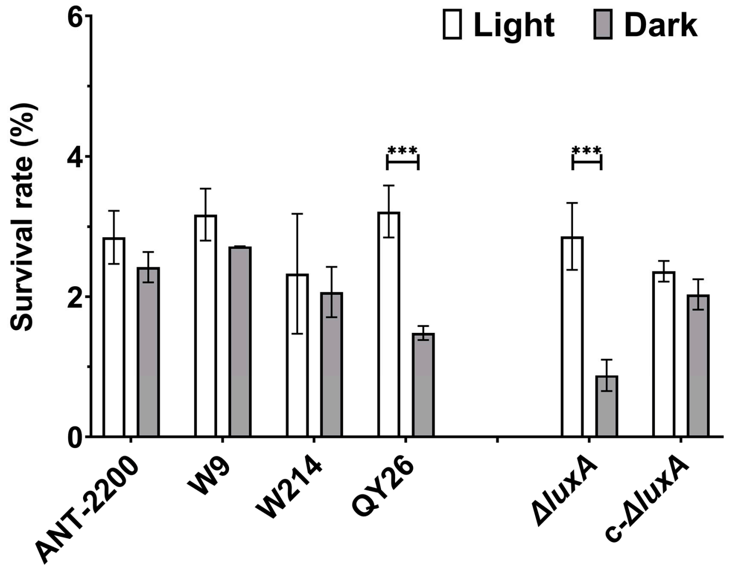

Figure S3. The influence of UV irradiation on different *Photobacterium* strains The survival rate of different strains in UV irradiation assay. The white and grey bars show the survival rate of cells cultured under light and dark conditions after UV irradiation, respectively. Asterisks indicate difference between different conditions at ***p < 0.001, (unpaired two-tailed Student's t test).

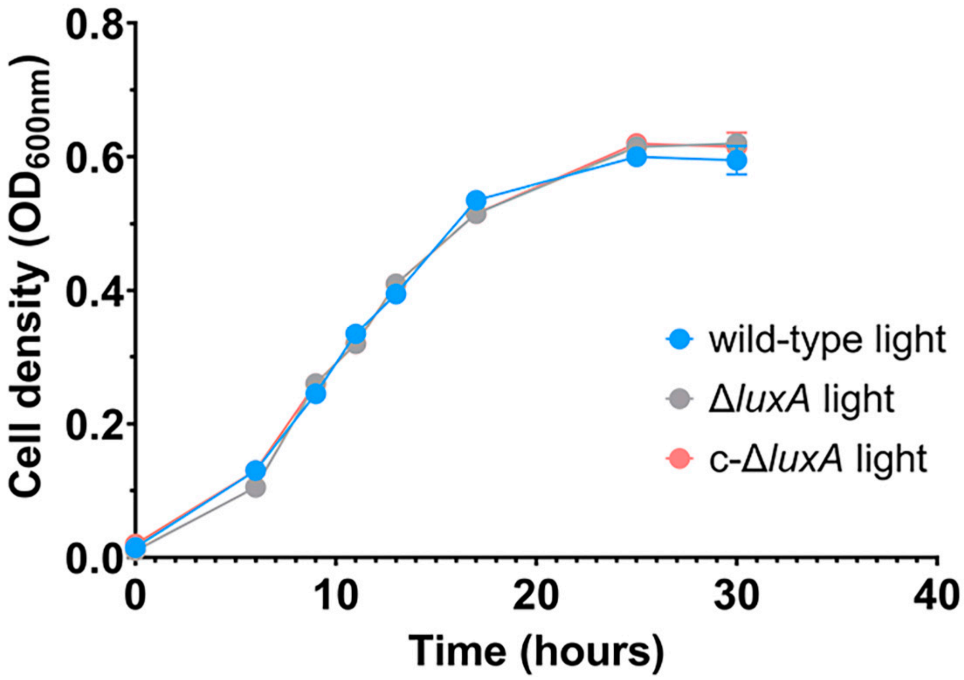

Figure S4. Growth recovery of wild-type strain, Δ*luxA* and c-Δ*luxA* with exposure to light after HHP treatment. The blue, gray and red lines represent wild-type strain, Δ*luxA* mutant and c-Δ*luxA* complementary strain, respectively.
